# Supplementary material for: Two gates mediate NMDA receptor activity and are under subunit-specific regulation
Source: Nat Commun. 2023 Mar 23;14:1623. doi: 10.1038/s41467-023-37260-y (PMC10036335; doi:10.1038/s41467-023-37260-y)
Supplement: Supplementary file 1 — Supplementary Information [file 41467_2023_37260_MOESM1_ESM.pdf]

SUPPLEMENTARY MATERIAL

Two gates mediate NMDA receptor activity and are under subunit-specific regulation

Amin, He et al

Supplementary Figure 1. Glycine-to-alanine substitutions at the conserved glycine in GluN1a or GluN2A reduce channel opening but in different ways (relates to Figure 2a-2i)... 2

Supplementary Table 1. Equilibrium analysis of single channel properties of wild-type GluN1a/GluN2A and G-to-A substitutions at conserved glycines (relates to Figure 2b-2c). ... 4

Supplementary Table 2. Cluster analysis of single channel properties of wild-type GluN1a/GluN2A and G-to-A substitutions at conserved glycines (relates to Figure 2b-2d).... 4

Supplementary Figure 2. Closed and open time histograms for wild-type GluN1a/GluN2A and G-to-A substitutions at the conserved glycines (relates to Figure 2e-2i)............................ 5

Supplementary Table 3. Closed state durations and occupancies for wild-type GluN1a/GluN2A and G-to-A substitutions at conserved glycines (relates to Figure 2e-2i). 6

Supplementary Table 4. Open state durations and occupancies for wild-type GluN1a/GluN2A and G-to-A substitutions at conserved glycines (relates to Figure 2e-2i). 7

Supplementary Figure 3. Molecular dynamic (MD) simulations of the transmembrane domain with the LBD-TMD linkers locked in a presumed open conformation (relates to Figure 3b-3e). 8

Supplementary Figure 4. Examples of ion trajectories (relates to Figure 3b-3e). 9

Supplementary Figure 5. Single channel activity in a NMDAR with the M3 gate locked 'open' by a tyrosine substitution (A650Y) in GluN2A (relates to Figure 4a-4d). 10

Supplementary Figure 6. Analysis of long-lived closed states for constructs with a tyrosine substitution (A650Y) at the M3 gate in GluN2A (relates to Figure 4a-4d & Supplementary Figure 5). 12

Supplementary Table 5. Equilibrium analysis of NMDARs containing GluN2A(A650Y) as well as G-to-A substitutions at conserved glycines (relates to Figure 4a-4d)...........................13

Supplementary Table 6. Cluster analysis of NMDARs containing GluN2A(A650Y) as well as G-to-A substitutions at conserved glycines (relates to Figure 4a-4d). 13

Supplementary Figure 7. Closed and open time histograms for GluN1/GluN2A(A650Y) alone or with G-to-A substitutions at conserved glycines (relates to Figure 4a-4d)..........................14

Supplementary Table 7. Closed state durations and occupancies for GluN1/GluN2A(A650Y) alone or with G-to-A substitutions at conserved glycines (relates to Figure 4a-4d)...............15

Supplementary Table 8. Open state durations and occupancies for GluN1/GluN2A(A650Y) alone or with G-to-A substitutions at conserved glycines (relates to Figure 4a-4d)...............16

Supplementary Table 9. Equilibrium analysis of NMDARs containing a cysteine in the external vestibule (GluN1a(V644C) and GluN2A(A650Y) as well as G-to-A substitutions at conserved glycines (relates to Figure 5e)...17

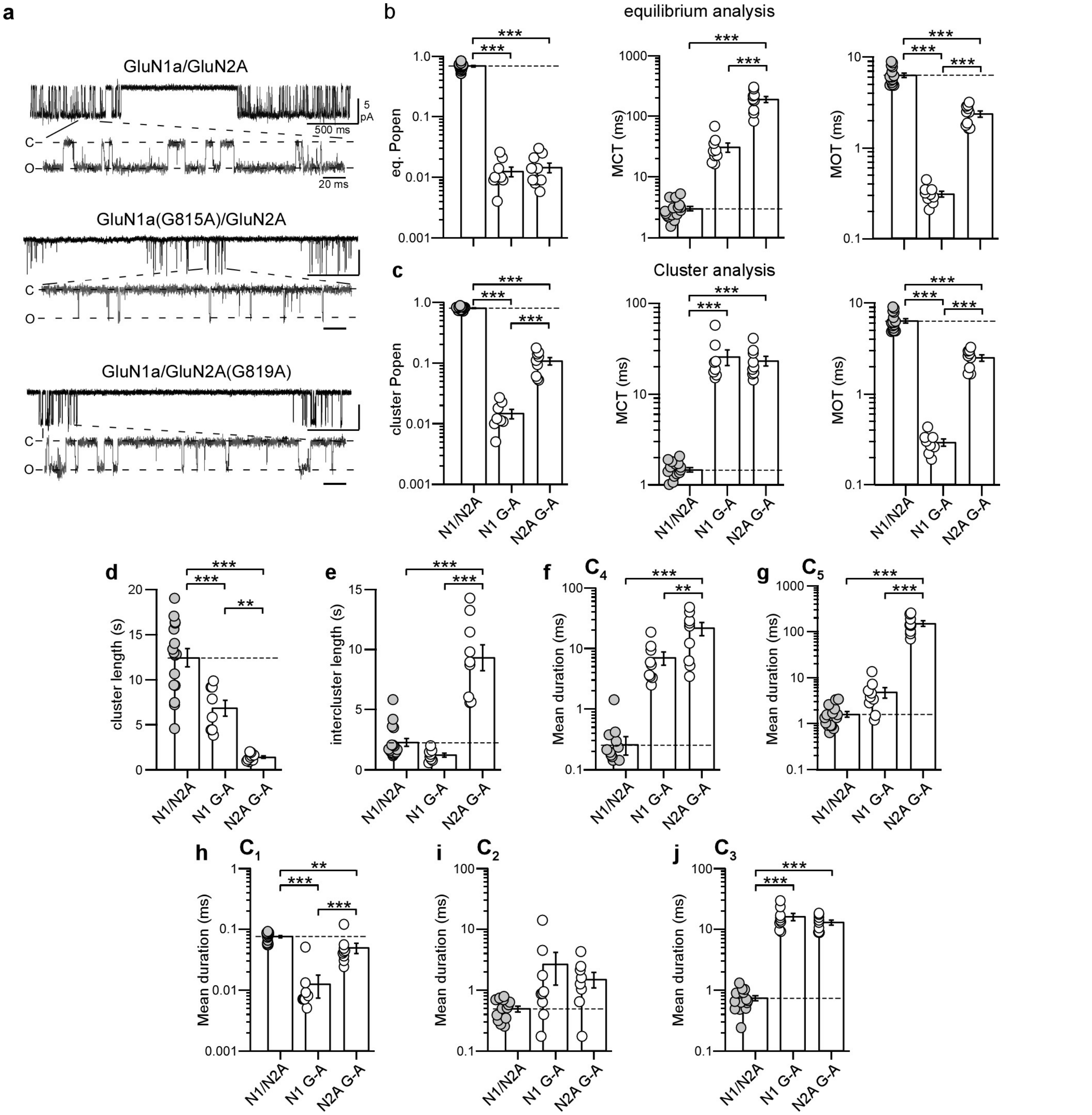

Supplementary Figure 1. Glycine-to-alanine substitutions at the conserved glycine in GluN1a or GluN2A reduce channel opening but in different ways (relates to Figure 2a-2i).

(a) Example single channel recordings of GluN1a/GluN2A and the same receptor containing alanine (A) substitutions at the conserved glycine (G) either in GluN1a(G815A) (N1 G-A) or GluN2A(G819A) (N2A G-A). Recordings were performed in the on-cell configuration (holding potential, +100 mV). Downward deflections are inward currents. For each construct, the top half shows a low-resolution (filtered at 1 kHz) and the bottom half a higher resolution portion of same record (3 kHz).

(b & c) Bar graphs (mean ± SEM with circles indicating individual values) showing single channel events assayed with (**b**) equilibrium analysis, including open probability (*eq. P_{open}*) (*left*), mean closed time (MCT) (*middle*), and mean open time (MOT) (*right*); or **(c)** cluster analysis, including

open probability (*cluster P_{open}*) **(*left*)**, mean closed time (MCT) (*middle*), and mean open time (MOT) (*right*). See Supplementary Tables 1 and 2 for number of replicates (ns).

(d & e) Bar graphs (mean ± SEM) for cluster (**d**) and inter-cluster (**e**) length. See Supplementary Table 2 for number of replicates (ns).

(f & g) Mean duration (mean ± SEM) for the two slowest components in the closed time histogram (Supplementary Figure 2), which are designated closed states C_4 (**f**) and C_5 (**g**). See Supplementary Table 3 for number of replicates (ns).

(h – j) Mean duration (mean ± SEM) for the three fastest components in the closed time histogram (Supplementary Figure 2), which are designated closed states C_1 (**h**), C_2 (**i**), and C_3 (**j**). See Supplementary Table 3 for number of replicates (ns).

***p < 0.01, ***p < 0.001, one-way ANOVA with post-hoc Tukey's test*. (**b, P_{open}**) *ANOVA* (*p = 8.2E-27*): wt vs N1 G-A, *p = 5.9E-15*; wt vs N2A G-A, *p = 5.8E-15*; N1 G-A vs N2A G-A, *p = 0.99*. (**b, MCT**) *ANOVA* (*p =4.6E-13*): wt vs N1 G-A, *p =0.21*; wt vs N2A G-A, *p = 4.7E-13*; N1 G-A vs N2A G-A, *p = 6.6E-10*. (**b, MOT**) *ANOVA* (*p = 1.8E-15*): wt vs N1 G-A, *p = 9.3E-15*; wt vs N2A G-A, *p = 8.7E-11*; N1 G-A vs N2A G-A, *p = 0.00023*.

(**c, P_{open}**) *ANOVA* (*p = 2.9E-29*): wt vs N1 G-A, *p = 4E-15*; wt vs N2A G-A, *p = 4E-15*; N1 G-A vs N2A G-A, *p = 0.0004*. (**c, MCT**) *ANOVA* (*p = 3.3E-08*): wt vs N1 G-A, *p = 4.5E-07*; wt vs N2A G-A, *p = 1.5E-06*; N1 G-A vs N2A G-A, *p = 0.82*. (**c, MOT**) *ANOVA* (*p = 4.2E-14*): wt vs N1 G-A, *p =8.4E-14*; wt vs N2A G-A, *p = 1.5E-09*; N1 G-A vs N2A G-A, *p = 0.0004*.

(**d**) *ANOVA* (*p = 7.7E-09*): wt vs N1 G-A, *p = 0.0007*; wt vs N2A G-A, *p = 4.9E-09*; N1 G-A vs N2A G-A, *p = 0.0033*. (**e**) *ANOVA* (*p = 3.5E-10*): wt vs N1 G-A, *p = 0.42*; wt vs N2A G-A, *p = 2.6E-09*; N1 G-A vs N2A G-A, *p = 3.5E-09*. (**f**) *ANOVA* (*p = 1.2E-05*): wt vs N1 G-A, *p = 0.17*; wt vs N2A G-A, *p = 6.9E-06*; N1 G-A vs N2A G-A, *p = 0.0036*. (**g**) *ANOVA* (*p = 1.2E-11*): wt vs N1 G-A, *p = 0.97*; wt vs N2A G-A, *p = 2.9E-11*; N1 G-A vs N2A G-A, *p = 6.5E-10*. (**h**) *ANOVA* (*p = 3.8E-08*): wt vs N1 G-A, *p = 2.0E-08*; wt vs N2A G-A, *p = 0.0068*; N1 G-A vs N2A G-A, *p = 0.0007*. (**i**) *ANOVA* (*p < 0.11*). (**j**) *ANOVA* (*p = 1.9E-10*): wt vs N1 G-A, *p =7.7E-10*; wt vs N2A G-A, *p = 9.6E-08*; N1 G-A vs N2A G-A, *p = 0.22*.

Both G-A mutants are significantly different from wild-type. In several notable instances (Cluster P_{open} & MOT and cluster & intercluster length), N1 G-A and N2A G-A are significantly different from each other.

Supplementary Table 1. Equilibrium analysis of single channel properties of wild-type GluN1a/GluN2A and G-to-A substitutions at conserved glycines (relates to Figure 2b-2c).

Construct	Total events (# of patches)	*i* *pA*	eq. P_{open}	MCT *ms*	MOT *ms*
N1/N2A	2,761,205 (16)	-6.7 ± 0.1	0.68 ± 0.02	3.0 ± 0.3	6.4 ± 0.4
N1(G815A)	783,410 (9)	-6.9 ± 0.4	0.012 ± 0.002	31 ± 5^	0.31 ± 0.02
N2A(G819A)	130537 (10)	-7.6 ± 0.5	0.015 ± 0.003	190 ± 20^	2.4 ± 0.2

Values shown are mean ± SEM for single-channel current amplitude (*i*), equilibrium open probability (eq. P_o), mean closed time (MCT), and mean open time (MOT). Single channel currents were recorded as in Figure 2a and Supplementary Figure 1 and analyzed in QuB (see Materials & Methods). Number of patches is in parenthesis below total events. Eq P_o is the fractional occupancy of the open states in the entire single-channel recording, including long lived closed states.

Supplementary Table 2. Cluster analysis of single channel properties of wild-type GluN1a/GluN2A and G-to-A substitutions at conserved glycines (relates to Figure 2b-2d).

Construct (# of patches analyzed)	T_{crit} *ms*	Cl. P_{open}	MCT *ms*	MOT *ms*	Cluster length *s*	Intercluster length *s*	$P_{cluster}$
N1/N2A (16)	91 ± 8	0.81 ± 0.01	1.5 ± 0.1	6.3 ± 0.3	12.3 ± 1.0	2.3 ± 0.3	0.84 ± 0.02
N1(G815A) (8)	540 ± 210	0.015 ± 0.003	25 ± 5	0.29 ± 0.03	6.8 ± 0.9	1.2 ± 0.2	0.84 ± 0.02
N2A(G819A) (9)	655 ± 170	0.11 ± 0.02	23 ± 3	2.5 ± 0.2	1.4 ± 0.1	9.3 ± 1.1	0.14 ± 0.02

Same data set as in Supplementary Table 1, but single channel records were analyzed using T_{Crit} (see Materials & Methods) to identify clusters. Values shown are mean ± SEM for T_{crit}, cluster open probability (Cl. P_o), mean closed time (MCT), mean open time (MOT), cluster length, intercluster length and $P_{cluster}$.

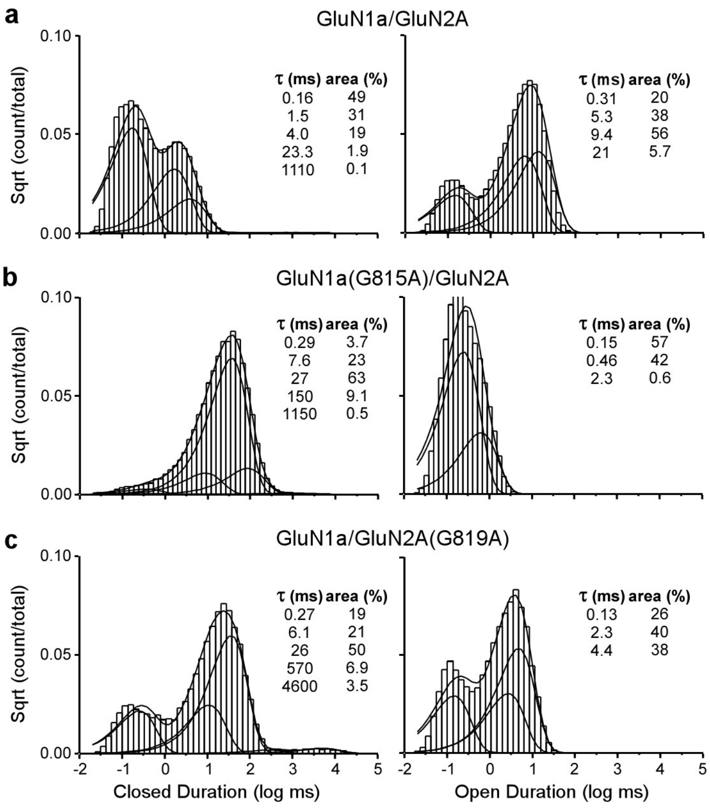

Supplementary Figure 2. Closed and open time histograms for wild-type GluN1a/GluN2A and G-to-A substitutions at the conserved glycines (relates to Figure 2e-2i).

(a-c) Closed (*left*) and open (*right*) time histograms for wild-type (**a**), GluN1a G-A (**b**), or GluN2A G-A (**c**). All closed time histograms were consistently best fit with 5 exponentials (Supplementary Table 3), whereas open time histograms were best fit typically with 4 (wild-type) or 3 (N1 G-A & N2A G-A) exponentials (Supplementary Table 4). Smooth lines are associated exponential fits.

Insets, mean closed and open state durations (τ, ms) and occupancies (α, %) (Supplementary Tables 3 & 4).

Supplementary Table 3. Closed state durations and occupancies for wild-type GluN1a/GluN2A and G-to-A substitutions at conserved glycines (relates to Figure 2e-2i).

Construct		C_1	C_2	C_3	C_4	C_5
**N1/N2A**	τ (ms)	0.16 ± 0.01	1.5 ± 0.1	4.0 ± 0.3	23.3 ± 3.1	1110 ± 110
	α (%)	49 ± 1	31 ± 2	19 ± 2	1.9 ± 1	0.14 ± 0.02
	Mean duration (ms)	0.07 ± 0.003	0.48 ± 0.05	0.74 ± 0.07	0.26 ± 0.09	1.6 ± 0.2
	n	15	15	15	15	15
**N1(G815A)**	τ (ms)	0.29 ± 0.05	7.6 ± 2.4	27 ± 6	150 ± 70	1150 ± 260
	α (%)	3.7 ± 0.6	24 ± 5	63 ± 3	9.1 ± 2.3	0.49 ± 0.1
	Mean duration (ms)	0.012 ± 0.005	2.6 ± 1.4	16 ± 2	6.9 ± 1.7	4.7 ± 1.2
	n	9	9	9	9	9
**N2A(G819A)**	τ (ms)	0.27 ± 0.10	6.1 ± 1.1	26 ± 3	570 ± 200	4640 ± 610
	α (%)	19 ± 1	21 ± 4	50 ± 3	6.9 ± 1.7	3.5 ± 0.5
	Mean duration (ms)	0.05 ± 0.01	1.5 ± 0.4	13 ± 1	21 ± 5	150 ± 21
	n	9	9	9	9	9

Mean values (± SEM) for closed state durations (τ, ms) and occupancies (α, %). Values were derived after fitting idealized single-channel records to a 5-closed and 3- to 4-open state kinetic scheme with optimal fits defined by log-likelihoods (see Materials & Methods).

Supplementary Table 4. Open state durations and occupancies for wild-type GluN1a/GluN2A and G-to-A substitutions at conserved glycines (relates to Figure 2e-2i).

Construct		O_1	O_2	O_3	O_4
**N1/N2A**	τ (ms)	0.31 ± 0.1	5.3 ± 0.9	9.4 ± 0.4	21 ± 2
	α (%)	20 ± 3	38 ± 9	56 ± 4	5.7 ± 2
	Mean duration (ms)	0.09 ± 0.05	3.0 ± 1	5.3 ± 0.5	1.1 ± 0.4
	n	15	15	9	9
**N1(G815A)**	τ (ms)	0.15 ± 0.01	0.46 ± 0.03	2.3 ± 0.6	
	α (%)	57 ± 2	42 ± 2	0.63 ± 0.3	
	Mean duration (ms)	0.09 ± 0.01	0.20 ± 0.02	0.007 ± 0.002	
	n	9	9	7	
**N2A(G819A)**	τ (ms)	0.13 ± 0.01	2.3 ± 0.4	4.4 ± 0.5	
	α (%)	26 ± 2	40 ± 7	38 ± 7	
	Mean duration (ms)	0.034 ± 0.003	1.1 ± 0.3	1.5 ± 0.2	
	n	9	9	8	

Mean values (± SEM) for open state durations (τ, ms) and occupancies (α, %). See legend to Supplementary Table 3 for details.

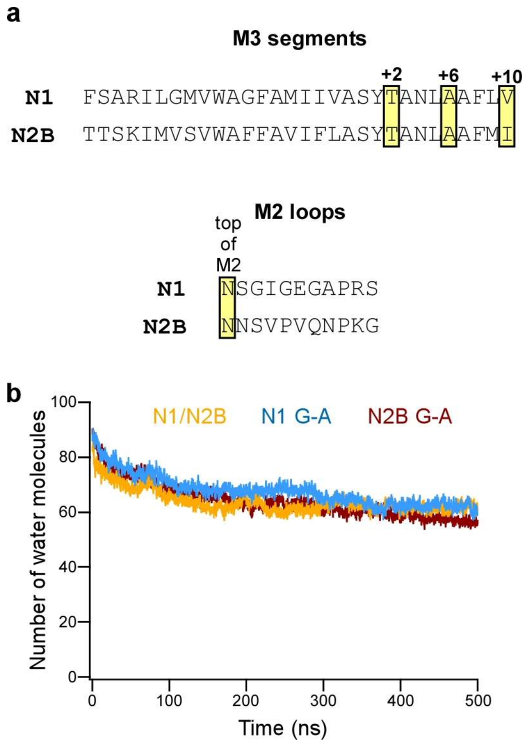

Supplementary Figure 3. Molecular dynamic (MD) simulations of the transmembrane domain with the LBD-TMD linkers locked in a presumed open conformation (relates to Figure 3b-3e).

(a) Schematic of GluN1 and GluN2B M3 segments and M2 loops indicating the positions used as references in the analysis of the MD simulations. Positions in the M3 segment are referenced to the serine (S), position ‘0’, in the highly conserved SYTANLAAF motif in M3.

(b) Number of water molecules in the pore during the MD simulation for the various constructs. Fifteen independent simulations each 500 ns long were run for each construct.

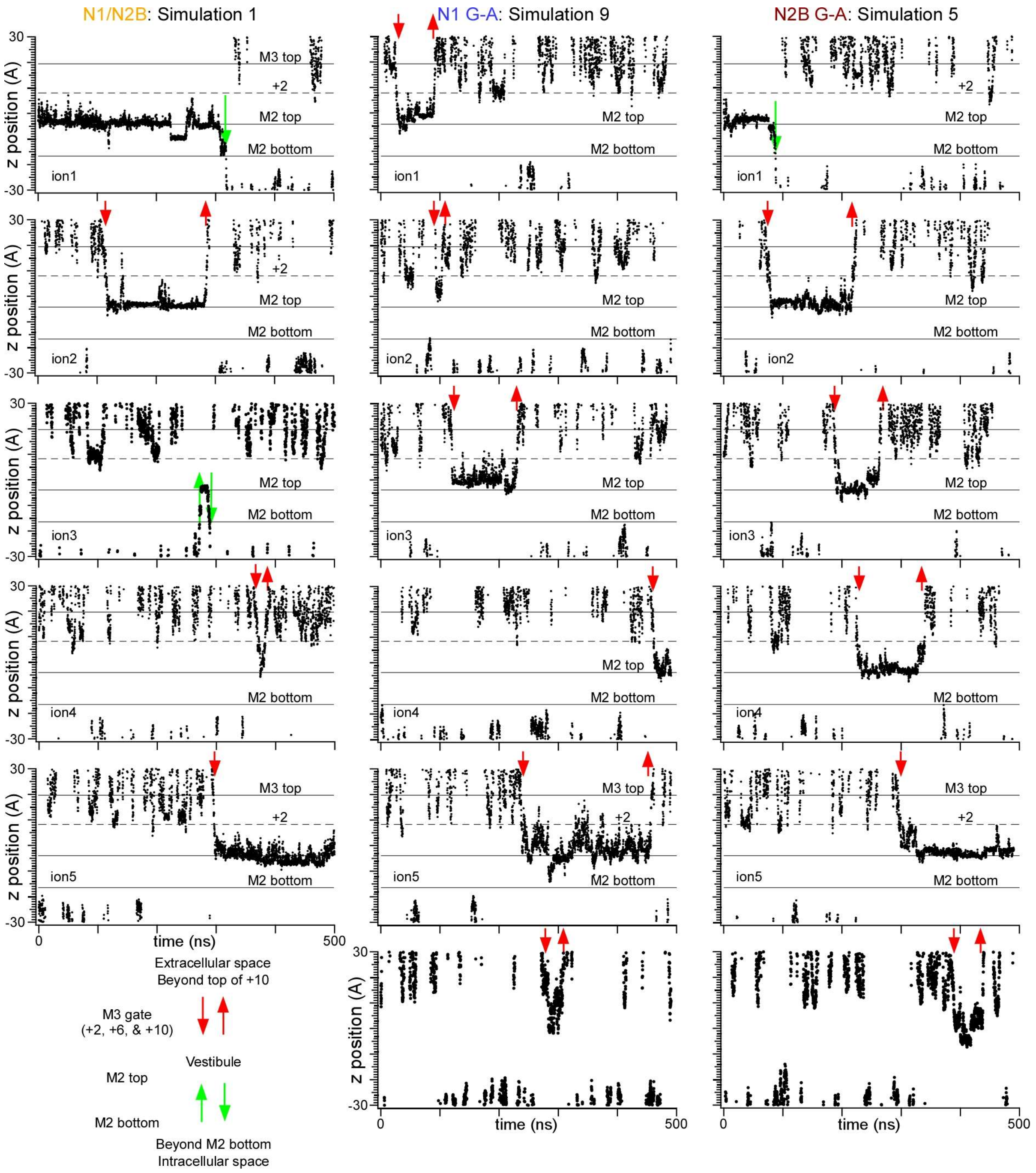

Supplementary Figure 4. Examples of ion trajectories (relates to Figure 3b-3e).
Full ion channel trajectories for GluN1/GluN2B simulation 1 (*left*), GluN1 G-A/GluN2B simulation 9 (*center*) or GluN1/GluN2B G-A simulation 5 (*right*). These simulations were selected for display since they showed the most crossings for that construct. Red arrows indicate complete crossings of the bundle helical crossings, whereas green arrows indicate complete crossings of the M2 pore loop (see Inset in bottom left corner).

Due to the periodic boundary condition in the simulations, the extracellular and intracellular space were connected, and so an ion leaving at z = 30 Å could reappear at z = -30 Å and vice versa.

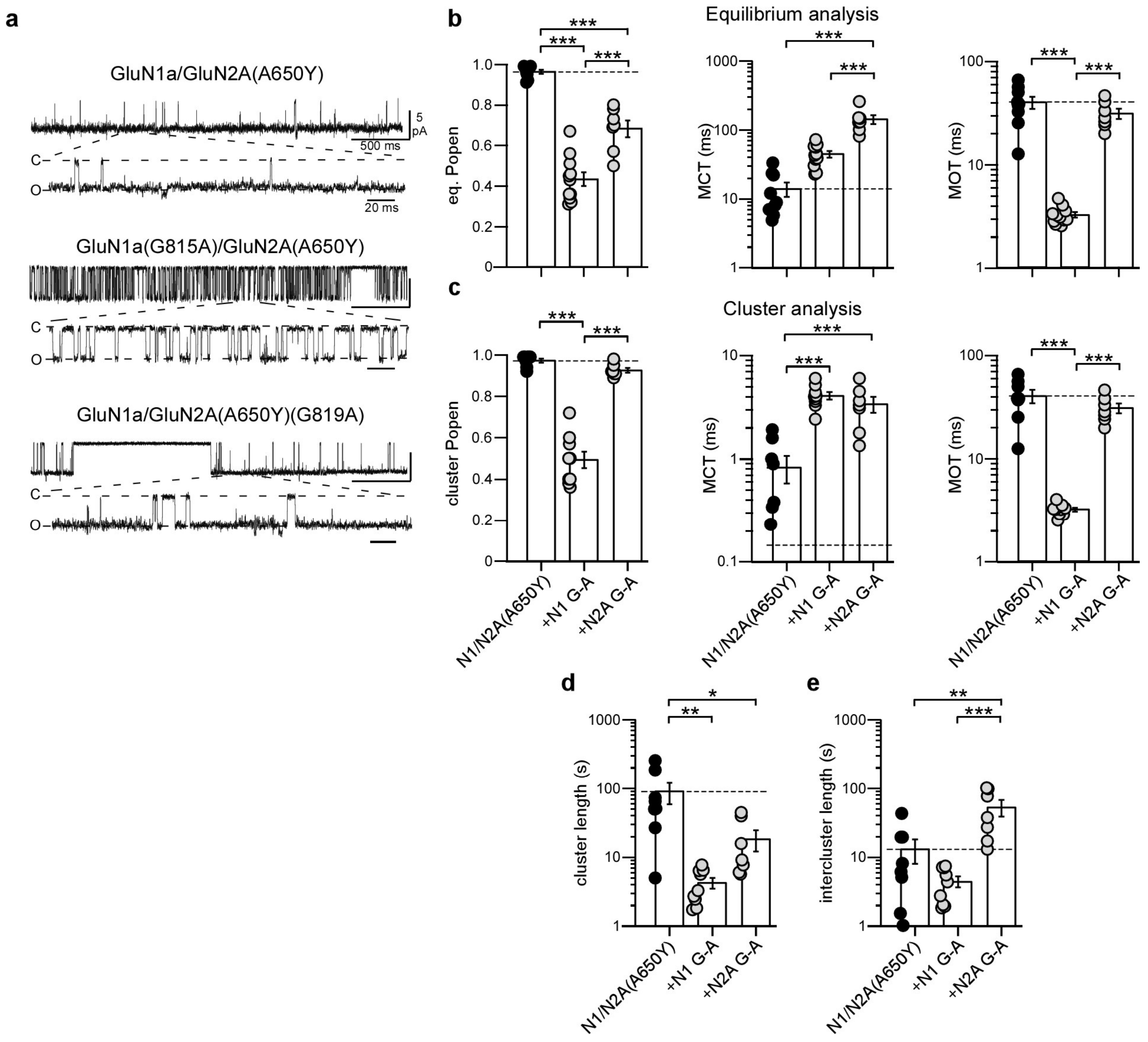

Supplementary Figure 5. Single channel activity in a NMDAR with the M3 gate locked 'open' by a tyrosine substitution (A650Y) in GluN2A (relates to Figure 4a-4d).

(a) Example single channel recordings of GluN1a/GluN2A(A650Y) alone or with the G-to-A substitutions at the conserved glycines in GluN1 or GluN2A. Recordings performed and displayed as in Supplementary Figure 1.

(b & c) Bar graphs (mean ± SEM) showing single channel events assayed with (**b**) equilibrium analysis, including open probability (*eq.* P_{open}) **(***left***)**, mean closed time (MCT) (*middle*), and mean open time (MOT) (*right*); or **(c)** cluster analysis, including open probability (*cluster* P_{open}) **(***left***)**, mean closed time (MCT) (*middle*), and mean open time (MOT) (*right*).

(d & e) Bar graphs (mean ± SEM) for cluster (**d**) and inter-cluster (**e**) length.

p < 0.05, **p < 0.01, ***p < 0.001, one-way ANOVA with post-hoc Tukey's test*. (b,** $\mathbf{P_{open}}$) *ANOVA* (*p = 2.5E-11*): A-Y vs N1 G-A, *p = 1.3E-11*; A-Y vs N2A G-A, *p = 1.1E-05*; N1 G-A vs N2A G-A, *p = 3.2E-05*. (**b, MCT**) *ANOVA* (*p = 5.0E-08*): A-Y vs N1 G-A, *p = 0.089*; A-Y vs N2A G-A, *p = 4.8E-08*; N1 G-A vs N2A G-A, *p = 2.3E-06*. (**b, MOT**) *ANOVA* (*p = 1.1E-07*): A-Y vs N1 G-A, *p = 1.4E-07*; A-Y vs N2A G-A, *p = 0.24*; N1 G-A vs N2A G-A, *p = 3.0E-05*.

(**c, P_{open}**) *ANOVA* (*p = 2.1E-11*): A-Y vs N1 G-A, *p = 7.0E-11*; A-Y vs N2A G-A, *p = 0.45*; N1 G-A vs N2A G-A, *p = 10E-10*. (**c, MCT**) *ANOVA* (*p = 1.9E-05*): A-Y vs N1 G-A, *p = 1.9E-05*; A-Y vs N2A G-A, *p = 0.0007*; N1 G-A vs N2A G-A, *p = 0.45*. (**c, MOT**) *ANOVA* (*p = 1.6E-06*): A-Y vs N1 G-A, *p = 1.8E-06*; A-Y vs N2A G-A, *p = 0.23*; N1 G-A vs N2A G-A, *p = 0.00015*.
(**d**) *ANOVA* (*p = 0.006*): A-Y vs N1 G-A, *p = 0.0066*; A-Y vs N2A G-A, *p = 0.035*; N1 G-A vs N2A G-A, *p = 0.84*. (**e**) *ANOVA* (*p = 0.0007*): A-Y vs N1 G-A, *p = 0.71*; A-Y vs N2A G-A, *p = 0.0056*; N1 G-A vs N2A G-A, *p = 0.0007*. See Supplementary Tables 5 & 6 for ns and additional parameters.

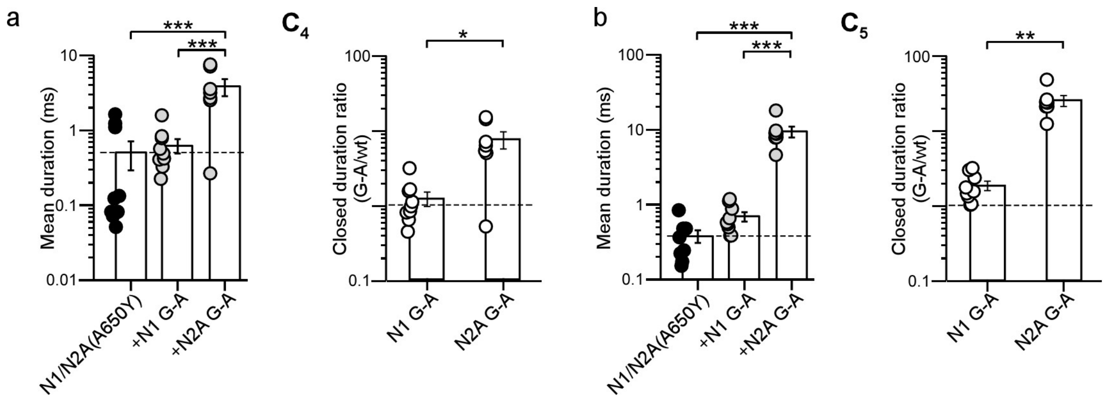

Supplementary Figure 6. Analysis of long-lived closed states for constructs with a tyrosine substitution (A650Y) at the M3 gate in GluN2A (relates to Figure 4a-4d & Supplementary Figure 5).

(a & b) Mean duration (mean ± SEM) (*left*) and ratio of mean durations between G-A constructs and reference (*right*) for the two slowest components in the closed time histogram (Supplementary Figure 7), which are designated closed states C_4 (**a**) and C_5 (**b**). The dashed is for wild-type.

p < 0.05, **p < 0.01, ***p < 0.001, one-way ANOVA with post-hoc Tukey's test* (*left panels*) *or two-tailed Student's t-test, unpaired* (*right panels*). (a, left**) *ANOVA* (*p = 0.00018*): A-Y vs N1 G-A, *p = 0.98*; A-Y vs N2A G-A, *p = 0.00039*; N1 G-A vs N2A G-A, p = 0.00059. (**b, left**) *ANOVA* (*p = 2.8E-08*): A-Y vs N1 G-A, *p = 0.95*; wt vs N2A G-A, *p = 9.7E-08*; N1 G-A vs N2A G-A, *p = 1.7E-07*. (***right panels***) *two-tailed Student's t-test, unpaired: p = 0.017* (**a**), *0.0014* (**b**). See Supplementary Tables 5 & 6 for ns and additional parameters.

Supplementary Table 5. Equilibrium analysis of NMDARs containing GluN2A(A650Y) as well as G-to-A substitutions at conserved glycines (relates to Figure 4a-4d).

Construct	Total events (# of patches)	*i* *pA*	eq. P_{open}	MCT *ms*	MOT *ms*
N1/ N2(A650Y)	279,315 (9)	-6.5 ± 0.2	0.96 ± 0.01	1.4 ± 0.3	40 ± 5
+N1(G815A)	2,876,786 (11)	-8.5 ± 0.5	0.44 ± 0.03	4.5 ± 0.5	3.2 ± 0.2
+N2A(G819A)	311,772 (7)	-6.9 ± 0.2	0.68 ± 0.04	14 ± 2	31 ± 4

Values shown are mean ± SEM for single-channel current amplitude (*i*), equilibrium open probability (eq. P_o), mean closed time (MCT), and mean open time (MOT). See Supplementary Table 1 for additional details.

Supplementary Table 6. Cluster analysis of NMDARs containing GluN2A(A650Y) as well as G-to-A substitutions at conserved glycines (relates to Figure 4a-4d).

Construct (# of patches analyzed)	T_{crit} *ms*	Cl. P_{open}	MCT *ms*	MOT *ms*	Cluster length *s*	Intercluster length *s*	$P_{cluster}$
N1/ N2A(A650Y) (8)	240 ± 100	0.97 ± 0.01	0.82 ± 0.23	40 ± 6	89 ± 31	1.3 ± 0.5	0.98 ± 0.01
+N1(G815A) (9)	100 ± 20	0.49 ± 0.04	4.1 ± 0.4	3.2 ± 0.1	4.2 ± 0.8	0.44 ± 0.08	0.90 ± 0.01
+N2A(G819A) (7)	1310 ± 440	0.92 ± 0.01	3.4 ± 0.6	31 ± 3	18 ± 6.3	5.2 ± 1.4	0.75 ± 0.04

Same data set as in Supplementary Table 5, but single channel records were analyzed using T_{Crit} (see Materials & Methods). Values shown are mean ± SEM for T_{crit}, cluster open probability (Cl. P_o), mean closed time (MCT), mean open time (MOT), cluster length, intercluster length and $P_{cluster}$.

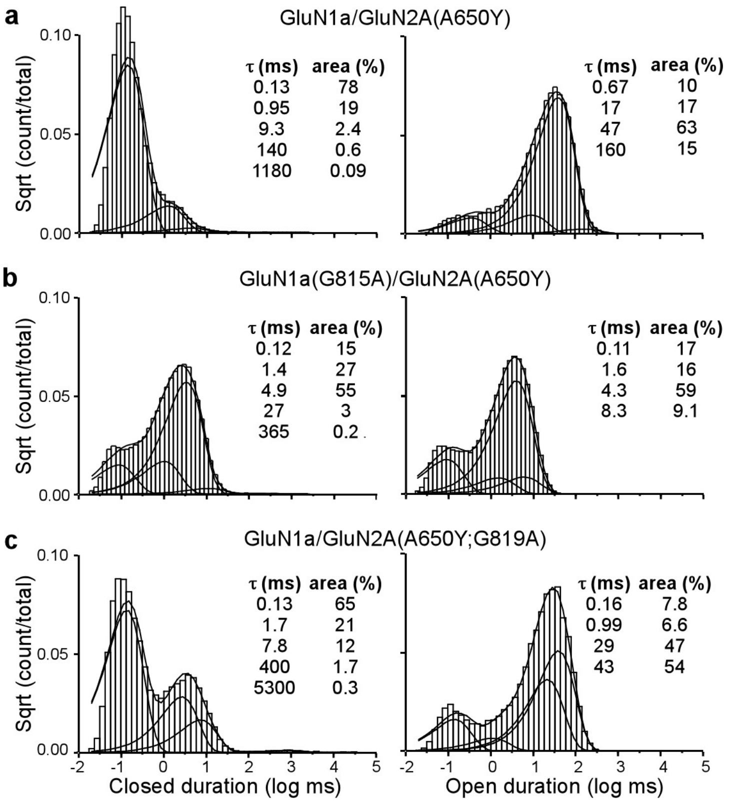

Supplementary Figure 7. Closed and open time histograms for GluN1/GluN2A(A650Y) alone or with G-to-A substitutions at conserved glycines (relates to Figure 4a-4d).

(a-c) Closed (*left*) and open (*right*) time histograms for GluN1/GluN2A(A650Y) (**a**), GluN1(G815A)/GluN2A(A650Y) (**b**), and GluN1/GluN2A(A650Y;G819A) (**c**). All closed time histograms were consistently best fit with 5 exponentials (Supplementary Table 7), and open time histograms by 4 exponentials (Supplementary Table 8). Smooth lines are associated exponential fits.

Insets, mean closed and open state durations (τ, ms) and occupancies (α, %) (Supplementary Tables 7 & 8).

Supplementary Table 7. Closed state durations and occupancies for GluN1/GluN2A(A650Y) alone or with G-to-A substitutions at conserved glycines (relates to Figure 4a-4d).

**Construct**		C_1	C_2	C_3	C_4	C_5
**N2A(A650Y)**	τ (ms)	0.13 ± 0.02	0.95 ± 0.1	9.3 ± 3.9	140 ± 90	1180 ± 490
	α (%)	78 ± 1	19 ± 8	2.4 ± 0.7	0.6 ± 0.2	0.09 ± 0.03
	Mean duration (ms)	0.09 ± 0.01	0.19 ± 0.09	0.20 ± 0.08	0.49 ± 0.21	0.38 ± 0.07
	n	9	9	9	9	9
**+N1(G815A)**	τ (ms)	0.12 ± 0.01	1.4 ± 0.2	4.9 ± 0.6	27 ± 6	365 ± 70
	α (%)	15 ± 1	27 ± 3	55 ± 3	3.0 ± 0.7	0.20 ± 0.03
	Mean duration (ms)	0.017 ± 0.002	0.41 ± 0.08	2.6 ± 0.3	0.61 ± 0.13	0.68 ± 0.10
	n	9	9	9	9	9
**+N2A(G819A)**	τ (ms)	0.13 ± 0.01	1.7 ± 0.3	7.8 ± 2.5	400 ± 130	5300 ± 1500
	α (%)	65 ± 3	21 ± 3	12 ± 2	1.7 ± 0.6	0.3 ± 0.1
	Mean duration (ms)	0.082 ± 0.004	0.39 ± 0.10	0.72 ± 0.08	3.8 ± 1	9.3 ± 1.5
	n	7	7	7	9	9

Mean values (± SEM) for closed state durations (τ, ms) and occupancies (α, %). Values were derived after fitting idealized single-channel records to a 4- to 5-closed and 2- to 4-open state kinetic scheme with optimal fits defined by log-likelihoods (see Materials & Methods). See Supplementary Table 3 for additional details.

Supplementary Table 8. Open state durations and occupancies for GluN1/GluN2A(A650Y) alone or with G-to-A substitutions at conserved glycines (relates to Figure 4a-4d).

Construct		O_1	O_2	O_3	O_4
**N2A(A650Y)**	τ (ms)	0.67 ± 0.13	17 ± 6	47 ± 8	160 ± 90
	α (%)	10 ± 2	17 ± 6	63 ± 10	15 ± 6
	Mean duration (ms)	0.08 ± 0.03	3.7 ± 1.9	31 ± 5	7.8 ± 2.5
	n	9	9	9	6
**+N1(G815A)**	τ (ms)	0.11 ± 0.01	1.6 ± 0.2	4.3 ± 0.2	8.3 ± 1.5
	α (%)	17 ± 1	16 ± 2	59 ± 3	9.1 ± 2.6
	Mean duration (ms)	0.02 ± 0.005	0.27 ± 0.06	2.5 ± 0.1	0.65 ± 0.18
	n	9	9	9	7
**+N2A(G819A)**	τ (ms)	0.16 ± 0.01	0.99 ± 0.07	29 ± 5	43 ± 7
	α (%)	7.8 ± 0.1	6.6 ± 0.8	47 ± 11	54 ± 10
	Mean duration (ms)	0.013 ± 0.002	0.065 ± 0.010	16 ± 5	21 ± 2
	n	7	7	7	5

Mean values (± SEM) for open state durations (τ, ms) and occupancies (α, %). See Supplementary Table 3 for details.

Supplementary Table 9. Equilibrium analysis of NMDARs containing a cysteine in the external vestibule (GluN1a(V644C) and GluN2A(A650Y) as well as G-to-A substitutions at conserved glycines (relates to Figure 5e).

Construct	Total events (# of patches)	*i* *pA*	eq. P_{open}	MCT *ms*	MOT *ms*
**N1(V644C)/ N2(A650Y)**	187,457 (5)	-7.4 ± 0.3	0.98 ± 0.005	0.46 ± 0.13	29.1 ± 1.8
**+N1(G815A)**	872,916 (4)	-9.6 ± 0.9	0.56 ± 0.02	3.5 ± 0.4	4.4 ± 0.5
**+N2A(G819A)**	78,403 (3)	-7.1 ± 0.1	0.67 ± 0.05	15.9 ± 4.3	30.3 ± 1.3

Values shown are mean ± SEM for single-channel current amplitude (*i*), equilibrium open probability (eq. P_o), mean closed time (MCT), and mean open time (MOT). See Supplementary Table 1 for additional details.
