# Supplementary material for: Two gates mediate NMDA receptor activity and are under subunit-specific regulation
Source: Nat Commun. 2023 Mar 23;14:1623. doi: 10.1038/s41467-023-37260-y (PMC10036335; doi:10.1038/s41467-023-37260-y)
Supplement: Supplementary file 3 — Reporting Summary [file 41467_2023_37260_MOESM3_ESM.pdf]

# nature portfolio

Corresponding author(s): Lonnie Wollmuth

Last updated by author(s): Feb 26, 2023



# Reporting Summary

Nature Portfolio wishes to improve the reproducibility of the work that we publish. This form provides structure for consistency and transparency in reporting. For further information on Nature Portfolio policies, see our Editorial Policies and the Editorial Policy Checklist.

## Statistics

For all statistical analyses, confirm that the following items are present in the figure legend, table legend, main text, or Methods section.

| n/a | Confirmed | |
|---|---|---|
| ☐ | ☒ | The exact sample size (*n*) for each experimental group/condition, given as a discrete number and unit of measurement |
| ☐ | ☒ | A statement on whether measurements were taken from distinct samples or whether the same sample was measured repeatedly |
| ☐ | ☒ | The statistical test(s) used AND whether they are one- or two-sided<br>*Only common tests should be described solely by name; describe more complex techniques in the Methods section.* |
| ☒ | ☐ | A description of all covariates tested |
| ☒ | ☐ | A description of any assumptions or corrections, such as tests of normality and adjustment for multiple comparisons |
| ☐ | ☒ | A full description of the statistical parameters including central tendency (e.g. means) or other basic estimates (e.g. regression coefficient) AND variation (e.g. standard deviation) or associated estimates of uncertainty (e.g. confidence intervals) |
| ☐ | ☒ | For null hypothesis testing, the test statistic (e.g. *F*, *t*, *r*) with confidence intervals, effect sizes, degrees of freedom and *P* value noted<br>*Give P values as exact values whenever suitable.* |
| ☒ | ☐ | For Bayesian analysis, information on the choice of priors and Markov chain Monte Carlo settings |
| ☒ | ☐ | For hierarchical and complex designs, identification of the appropriate level for tests and full reporting of outcomes |
| ☒ | ☐ | Estimates of effect sizes (e.g. Cohen's *d*, Pearson's *r*), indicating how they were calculated |

*Our web collection on statistics for biologists contains articles on many of the points above.*

## Software and code

Policy information about availability of computer code

| | |
|---|---|
| Data collection | All patch clamp data were recorded using HEKA PatchMaster v2x90.2. Associated information for data collection, e.g., sampling rate, is included in Methods section. |
| Data analysis | Custom code/software was not required for this study. Standard software packaged within Igor Pro v7, Microsoft Excel 365, QuB, and CHARMM-GUI. The availability and/or source of the software is indicated in the Methods section. |

For manuscripts utilizing custom algorithms or software that are central to the research but not yet described in published literature, software must be made available to editors and reviewers. We strongly encourage code deposition in a community repository (e.g. GitHub). See the Nature Portfolio guidelines for submitting code & software for further information.

## Data

Policy information about availability of data

All manuscripts must include a data availability statement. This statement should provide the following information, where applicable:

- Accession codes, unique identifiers, or web links for publicly available datasets
- A description of any restrictions on data availability
- For clinical datasets or third party data, please ensure that the statement adheres to our policy

A full Data Availability statement is provided in the main manuscript.

## Human research participants

Policy information about studies involving human research participants and Sex and Gender in Research.

| | |
|---|---|
| Reporting on sex and gender | N/A |
| Population characteristics | N/A |
| Recruitment | N/A |
| Ethics oversight | N/A |

Note that full information on the approval of the study protocol must also be provided in the manuscript.

# Field-specific reporting

Please select the one below that is the best fit for your research. If you are not sure, read the appropriate sections before making your selection.

☒ Life sciences ☐ Behavioural & social sciences ☐ Ecological, evolutionary & environmental sciences

For a reference copy of the document with all sections, see nature.com/documents/nr-reporting-summary-flat.pdf

# Life sciences study design

All studies must disclose on these points even when the disclosure is negative.

| | |
|---|---|
| Sample size | No statistical test to define sample size was used. Rather, because our experiments are biophysical in nature, we based sample size on previous publications. |
| Data exclusions | In most instances, single channel recordings were included inly if they had greater than 5000 (most had >50,000). In a few instances where the construct showed a low Popen, we would include a cell with fewer events. |
| Replication | Samples were replicated at least an n of 3 for all experiments. For MD simulations we carried out a minimum of 8 (often 12-16) independent replications for each set of MD simulations. Infomration on the number of replication for each manipulation are included in the Methods and Figure legends. |
| Randomization | On any recording day we would record, if possible, 1 wild type construct, 1 GluN1 G-A and 1 GluN2A G-A in random order. |
| Blinding | Blinding was not required in this study since experimental outcomes were robust and quantification was independent of the user. |

# Reporting for specific materials, systems and methods

We require information from authors about some types of materials, experimental systems and methods used in many studies. Here, indicate whether each material, system or method listed is relevant to your study. If you are not sure if a list item applies to your research, read the appropriate section before selecting a response.

## Materials & experimental systems

| n/a | Involved in the study | |
|---|---|---|
| ☒ | ☐ | Antibodies |
| ☐ | ☒ | Eukaryotic cell lines |
| ☒ | ☐ | Palaeontology and archaeology |
| ☒ | ☐ | Animals and other organisms |
| ☒ | ☐ | Clinical data |
| ☒ | ☐ | Dual use research of concern |

## Methods

| n/a | Involved in the study | |
|---|---|---|
| ☒ | ☐ | ChIP-seq |
| ☒ | ☐ | Flow cytometry |
| ☒ | ☐ | MRI-based neuroimaging |

## Eukaryotic cell lines

Policy information about cell lines and Sex and Gender in Research

| | |
|---|---|
| Cell line source(s) | Human embryonic kidney 293 (HEK 293) cells (CRL-1573TM) are obtained from ATCC® approximately every two years. Multiple aliquots of these stocks are made and frozen down in liquid nitrogen. Approximately once per month, we thaw one |

| | |
|---|---|
| | of these aliquots and use for the subsequent time period. |
| Authentication | Cells derived from these aliquots display highly consistent morphology and growth patterns. If we have concerns about our stocks of HEK 293 cells, we obtain new stocks from ATCC®. |
| Mycoplasma contamination | A PCR-based mycoplasma detection kit is regularly employed to detect mycoplasma in cell cultures. All PCR tests were negative for mycoplasma. |
| Commonly misidentified lines (See ICLAC register) | None |
